# Case Report: A Case of Cystoid Macular Edema in Retinitis Pigmentosa With Central Retinal Vein Occlusion

**DOI:** 10.3389/fmed.2022.877429

**Published:** 2022-06-09

**Authors:** Da-Hu Wang, Cao Gu, Yuan-Zhi Yuan

**Affiliations:** ^1^Department of Ophthalmology, Longhua Hospital Affiliated to Shanghai University of Traditional Chinese Medicine, Shanghai, China; ^2^Department of Ophthalmology, Shanghai Xinshijie Zhongxing Eye Hospital, Shanghai, China; ^3^Department of Ophthalmology, Zhongshan Hospital Affiliated to Fudan University, Shanghai, China; ^4^Centre for Evidence-Based Medicine, Fudan University, Shanghai, China

**Keywords:** cystoid macular edema, retinitis pigmentosa (RP), central retinal vein occlusion (CRVO), anti-VEGF (vascular endothelial growth factor), case report

## Abstract

**Significance:**

Cystoid macular edema (CME) is a common complication of retinitis pigmentosa (RP). However, CME in RP with central retinal vein occlusion (CRVO) is rare. Prompt administration of anti-vascular endothelial growth factor (anti-VEGF) medication can achieve a satisfactory prognosis.

**Purpose:**

This report describes a case of using anti-VEGF medication to treat CME secondary to RP with impending or mild CRVO.

**Case Report:**

A 26-year-old female presented for blurred vision in both eyes. Best-corrected visual acuity (BCVA) was 20/50 in the right eye and finger-counting in the left eye. According to ophthalmic examinations, CME secondary to RP in the right eye and CME secondary to RP with impending or mild CRVO in her left eye can be diagnosed. Central macular thickness (CMT) was 554 μ m in the right eye and 831 μm in the left eye. Only the left eye was treated with a single intravitreal injection of anti-VEGF medication. One month later, BCVA increased to 20/200 and CMT decreased to 162 μm in the left eye. Interestingly, BCVA in the right eye also had an improvement (20/40) and intraretinal fluid decreased significantly. However, 3 months after injection, these improvements of both eyes were not maintained.

**Conclusion:**

This is the second case of RP with CRVO. Intravitreal injection of anti-VEGF medication for addressing CME secondary to RP with CRVO is an effective treatment, but it needs to be reinjected.

## Introduction

Retinitis pigmentosa (RP) is a heterogeneous group of inherited degenerative retinal disorders that result in loss of rod and cone photoreceptor cells and dystrophy of the pigment epithelium (1). About 20–30 percent of RP patients have associated systemic diseases, such as Usher’s syndrome and Bardet-Biedl syndrome (2). The disease is characterized by night blindness and peripheral visual field defects, resulting in noticeable tunnel vision and legal blindness in the late stage of the disease. Haim (3) reported that RP had a prevalence of approximately 1/4,000 with a variable age of onset (from adolescence to adulthood). Vision loss occurs in RP patients through progressive loss of photoreceptors and development of complications such as cystoid macular edema (CME), epiretinal membrane, macular hole, vitreomacular traction syndrome and cataract (2, 4, 5). Currently, there is no effective treatment delaying or reversing photoreceptor degeneration, but treatment of these complications may improve vision (6–8).

CME is a common complication of the disease occurring in 8.0–58.6% of patients based on clinic surveys (4, 5, 9). So far, the pathogenesis of CME in RP is not clear. Dysfunction of Muller cell osmoregulation, degeneration/dysregulation of the retinal pigment epithelium, destruction of the blood-retinal barrier, and vitreoretinal traction had been implicated (6). Visual acuity in RP patients is mostly deteriorated by CME, so treatment should be considered, even if the underlying disorder itself continues to progress with an overall poor long-term prognosis. At present, CME have been managed in different ways, such as carbonic anhydrase inhibitors (CAIs), corticosteroids, laser photocoagulation, anti-vascular endothelial growth factor (anti-VEGF) medications and pars plana vitrectomy (7).

In addition, there are some uncommon ocular comorbidities in RP patients, such as central retinal vein occlusion (CRVO) and Coats-like exudative vitreoretinopathy (10, 11). Therefore, CME in RP with CRVO is extremely rare. In the context of the studies with intravitreal anti-VEGF drugs for the management of CME secondary to RP, we present a case of CME in RP with CRVO treated with aflibercept.

## Case Report

### Initial Examination

**FIGURE 1:**
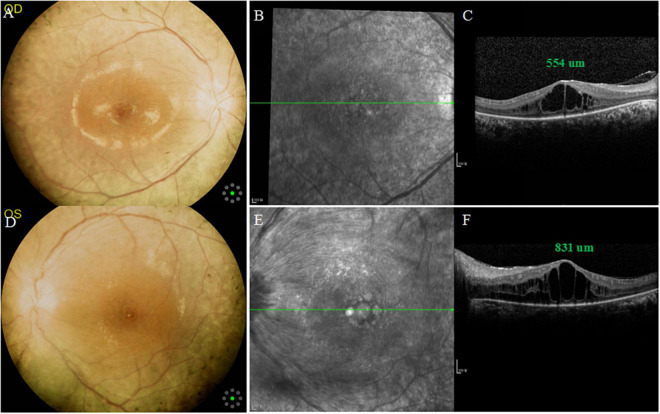
Multimodal imaging of both eyes at the initial examination. **(A,D)** Color fundus photographs showing arteriolar narrowing, cystoid macular edema (CME) and mid-peripheral bone-spicule-like pigmentations in both eyes, and tortuous and dilated superior temporal vein in the left eye. **(B,E)** Infrared fundus photographs showing CME in both eyes, and swelling of retinal nerve fiber layer in the left eye. **(C,F)** SD-OCT: central macular thickness (CMT) was 554 μm in the right eye and 831 um in the left eye.

**FIGURE 2:**
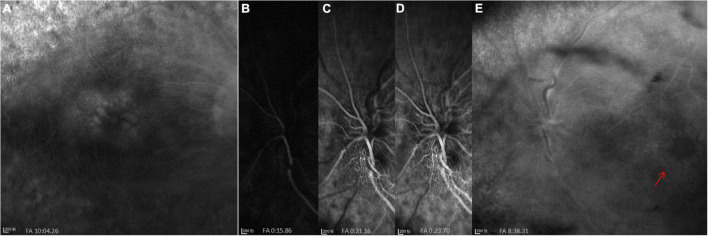
FFA images at initial visit. **(A)** Cystoid macular edema (CME) in the right eye. **(B–E)** A significant filling delay of the main veins (especially the superior veins) and CME (red arrow) in the left eye.

**FIGURE 3:**
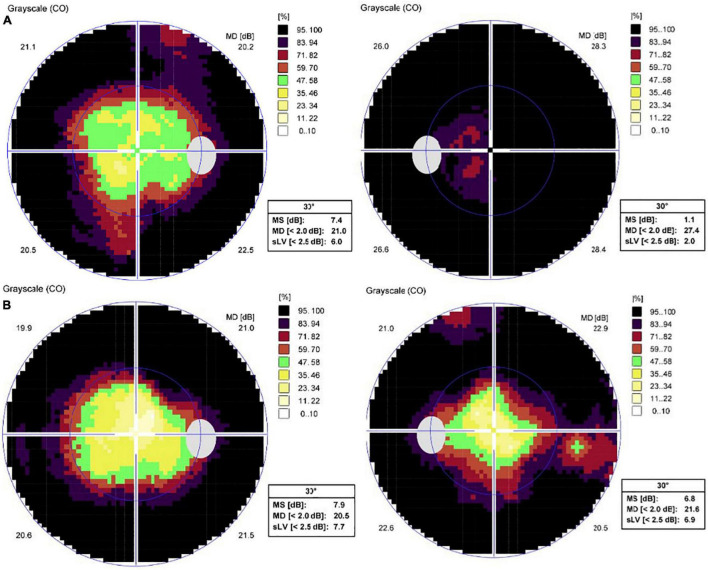
Standard automated perimetry test results of both eyes. **(A)** Peripheral visual field defects of both eyes at the initial examination. **(B)** One month after intravitreal aflibercept in the left eye, visual field of the left eye improved.

A 26-year-old female with no remarkable past medical and family history came to the outpatient department of the Department of Ophthalmology, Longhua Hospital Affiliated to Shanghai University of Traditional Chinese Medicine complaining for blurred vision in her left eye. Upon examination, her best-corrected visual acuity (BCVA) was 20/50 in the right eye and finger-counting in the left eye. Intraocular pressure (IOP) was 15 mmHg in the right eye and 16 mmHg in the left eye. Anterior segment examination of both eyes was unremarkable, and a mild posterior subcapsular opacification of crystalline lens was observed with a left relative afferent pupillary defect (RAPD). Color fundus photography showed the arteriolar narrowing, CME and mid-peripheral hyperpigmentary spots in form of bone-spiculesand in both eyes (Figure 1), and the tortuous and dilated superior temporal vein in the left eye. Spectral Domain Optical coherence tomography (SD-OCT) (Spectralis OCT, Heidelberg Engineering, Germany), fundus fluorescein angiography (FFA; Heidelberg Retina Angiograph, Heidelberg Engineering, Germany) and visual field tests with Octopus 900 (TOP strategy) were also performed, as shown in Figures 1–3.

SD-OCT scan showed that central macular thickness (CMT) was 554 μm in the right eye and 831 μm in the left eye (Figure 1). FFA showed the normal arm-retinal arteria filling time (15.8 s) and a significant filling delay of the main veins (23.7 s) in the left eye, and CME in both eyes (Figure 2). Moreover, the perimetry showed a noticeable tunnel vision in both eyes, especially in the left eye (Figure 3).

In addition, a panel of medical examinations were also performed including a chest X-ray, arterial blood pressure (BP), complete blood cell count (CBC), blood sugar level, hemoglobin A1c level (HbA1c), low-density lipoprotein cholesterol (LDL-C), homocysteinemia level, and the detection of parameter of inflammatory and infectious diseases. No systemic diseases including systemic vasculitis were found.

Based on above inspections, the patient was clinically diagnosed with CME secondary to RP in the right eye, and CME in RP with impending or mild CRVO in her left eye, although genetic testing and electroretinogram (ERG) were not performed. It should be emphasized that she was informed about the off-label use of aflibercept and signed informed consent before treatment.

### Follow-Up Visits

**FIGURE 4:**
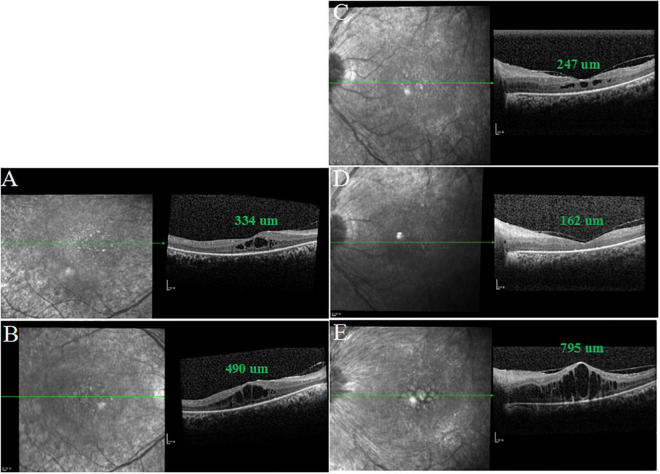
SD-OCT images of both eyes after intravitreal injection of aflibercept in the left eye. **(A,D)** One month after treatment, CMT was 334 μm in the right eye and macular edema (ME) of the left eye completely disappeared. **(C)** One week after treatment, CMT was 247 μm in the left eye. **(B,E)** Three months after treatment, CMT was 490 μm in the right eye and ME of the left eye relapsed.

One week after one dose of intravitreal aflibercept (2 mg/0.05 ml Eylea^^®^^; Bayer AG, Leverkusen, Germany) in the left eye, OCT scan showed a significant decrease in CMT (247 μm) (Figure 4) and BCVA elevated to 20/400. One month after treatment, BCVA in the left eye elevated to 20/200, macular edema (ME) completely disappeared and the visual field (VF) also improved accordingly (Figures 3, 4). Interestingly, BCVA in the right eye also had an improvement (20/40) and intraretinal fluid decreased significantly. However, 3 months after injection, BCVA and CMT of both eyes were not maintained.

## Discussion

CME is a common complication of RP, but the mechanisms are unknown. It should be emphasized that treatment should be considered only if CME is excessive and disrupts the central vision in patients with impaired peripheral vision (7). Up to date, although there is not a standard approach for CME secondary to RP, people are still working on it. A recent systematic review recommends oral CAIs as the first line treatment in CME secondary to RP (12). But considering the side effects of these drugs, CAIs are not suitable for the pediatric and elderly patients (13). A prospective, non-randomized comparative trial demonstrated that intravitreal injection of triamcinolone acetonide (4 mg/0.1 ml) could obtain a good anatomical result in patients with CME secondary to RP, but no statistically significant changes were observed in BCVA between the two groups (14). In addition, according to reports in the literature (12), anti-VEGF agents can treat CME secondary to RP with good results, although none of these drugs have been approved for this indication yet.

So far, only one case of RP with ischemic CRVO has been reported (10). As we know, retinal vein occlusion (RVO) is the second most common retinal vascular disease (15). The prevalence rate of CRVO is 0.08% in patients aged 30 years and older (16). Its exact pathogenesis is still completely unclear. Virchow’s triad-vessel damage, stasis and blood hypercoagulability may be related to the disease (15). Nevertheless, the role of thrombophilic risk factors in younger patients with RVO is still controversial. ME is the leading cause of vision loss in RVO (17). At present, intravitreal anti-VEGF agents have been developed as the front-line therapy for ME secondary to RVO (18). Therefore, we used aflibercept to treat CME secondary to RP with CRVO in this case.

Our results showed that 1 week after a single intravitreal injection, CMT in the left eye significantly decreased from 831 to 247 μm and BCVA elevated to 20/400. One month after treatment, ME in the left eye completely disappeared and CMT was 162 um. Accordingly, BCVA and VF also improved. This could be explained by the fact that ME deteriorated visual function in RP patients. Interestingly, BCVA and ME in the untreated eye also had an improvement (Figure 4). The most likely explanation is that the systemic absorption of the drug affects the fellow eye (19). However, some studies provided conflicting conclusions (20, 21). Three months after injection, BCVA and CMT in both eyes were not maintained, which might further suggest the possibility of systemic absorption of intravitreal anti-VEGF drugs. At this point, the patient refused a second dose of anti-VEGF agent in the left eye. Therefore, further studies are needed to investigate the management of rebound CME secondary to RP with CRVO.

## Conclusion

This extremely rare case describes the clinical features of RP with impending or mild CRVO. Moreover, intravitreal injection of anti-VEGF drug is efficacious for addressing CME secondary to RP with CRVO, but it needs to be reinjected.

## Data Availability Statement

The original contributions presented in the study are included in the article/supplementary material, further inquiries can be directed to the corresponding author.

## Ethics Statement

Written informed consent was obtained from the individual(s) for the publication of any potentially identifiable images or data included in this article.

## Author Contributions

D-HW carried out the collection of the reported studies and the acquisition of data, with input from CG. D-HW conceived the study, made the interpretation of the information, and drafted the initial manuscript. Y-ZY reviewed and revised the manuscript. All co-authors read and approved the final manuscript.

## Conflict of Interest

The authors declare that the research was conducted in the absence of any commercial or financial relationships that could be construed as a potential conflict of interest.

## Publisher’s Note

All claims expressed in this article are solely those of the authors and do not necessarily represent those of their affiliated organizations, or those of the publisher, the editors and the reviewers. Any product that may be evaluated in this article, or claim that may be made by its manufacturer, is not guaranteed or endorsed by the publisher.
